# Supplementary material for: Potential Anti-Alzheimer Properties of Mogrosides in Vitamin B12-Deficient Caenorhabditis elegans
Source: Molecules. 2023 Feb 15;28(4):1826. doi: 10.3390/molecules28041826 (PMC9961707; doi:10.3390/molecules28041826)
Supplement: Supplementary file 1 [file molecules-28-01826-s001.zip › molecules-2152552-supplementary.pdf]

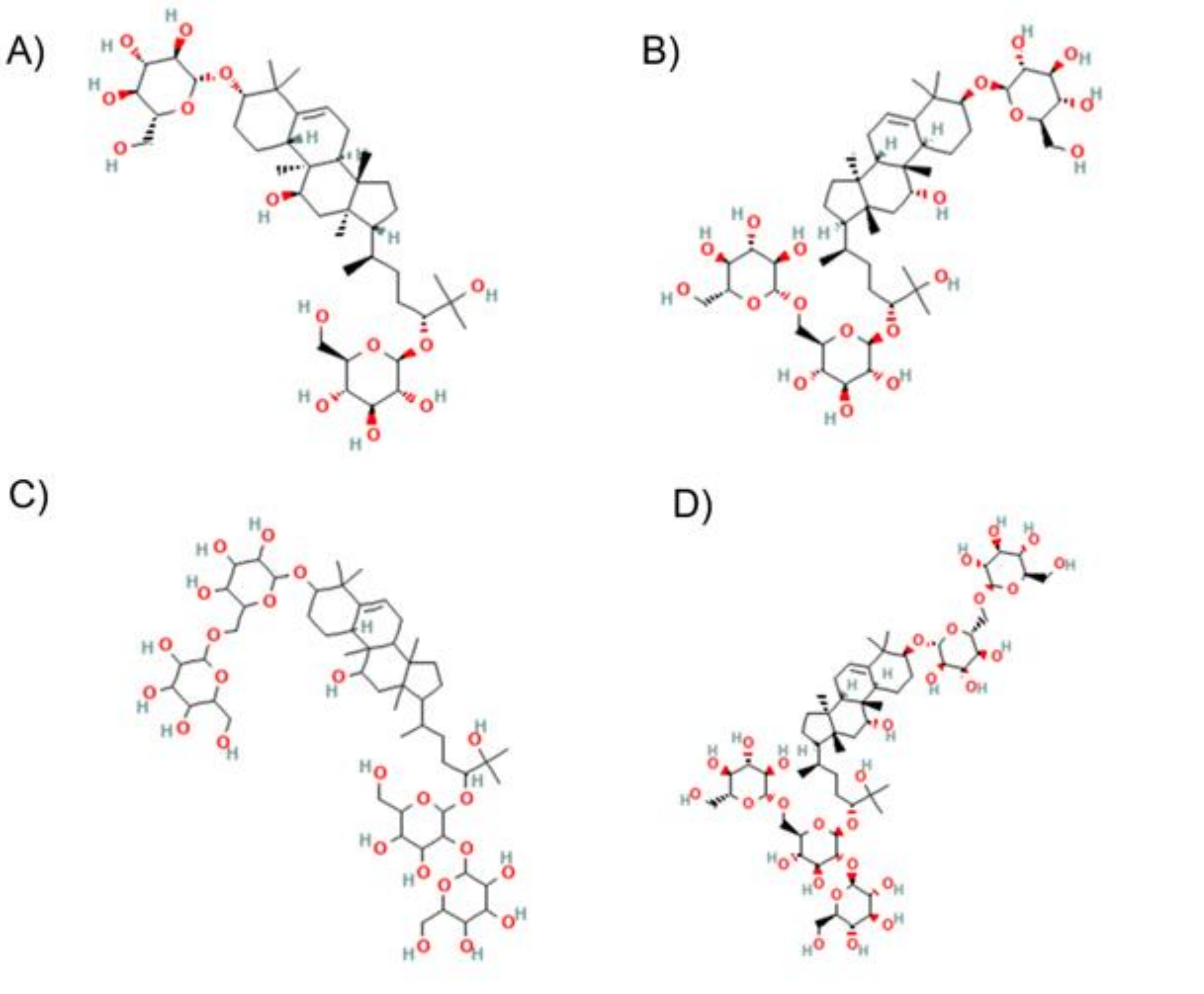

Figure S1: Mogrosides Structures. (A) Mogroside IIe; (B) Mogroside III; (C) Mogroside IV; (D) Mogroside V. Source of mogrosides structures: https://pubchem.ncbi.nlm.nih.gov

Table S1: Molecular weight of mogrosides IIe, III, IV and V. https://pubchem.ncbi.nlm.nih.gov.

Compound Name	Molecular Weight	PubChem CID
Mogroside IIe	801.0 g/mol	24721558
Mogroside III	963.2 g/mol	24720988
Mogroside IV	1125.3 g/mol	14525324
Mogroside V	1287.4 g/mol	24721270
